# Isoflurane-Induced Spatial Memory Impairment in Mice is Prevented by the Acetylcholinesterase Inhibitor Donepezil

**DOI:** 10.1371/journal.pone.0027632

**Published:** 2011-11-17

**Authors:** Diansan Su, Yanxing Zhao, Beilei Wang, Huan Xu, Wen Li, Jie Chen, Xiangrui Wang

**Affiliations:** Department of Anesthesiology, Renji Hospital, School of Medicine, Shanghai Jiaotong University, Shanghai, China; Pontifical Catholic University of Rio Grande, Brazil

## Abstract

Although many studies have shown that isoflurane exposure impairs spatial memory in aged animals, there are no clinical treatments available to prevent this memory deficit. The anticholinergic properties of volatile anesthetics are a biologically plausible cause of cognitive dysfunction in elderly subjects. We hypothesized that pretreatment with the acetylcholinesterase inhibitor donepezil, which has been approved by the Food and Drug Administration (FDA) for the treatment of Alzheimer's disease, prevents isoflurane-induced spatial memory impairment in aged mice. In present study, eighteen-month-old mice were administered donepezil (5 mg/kg) or an equal volume of saline by oral gavage with a feeding needle for four weeks. Then the mice were exposed to isoflurane (1.2%) for six hours. Two weeks later, mice were subjected to the Morris water maze to examine the impairment of spatial memory after exposure to isoflurane. After the behavioral test, the mice were sacrificed, and the protein expression level of acetylcholinesterase (AChE), choline acetylase (ChAT) and α7 nicotinic receptor (α7-nAChR) were measured in the brain. Each group consisted of 12 mice. We found that isoflurane exposure for six hours impaired the spatial memory of the mice. Compared with the control group, isoflurane exposure dramatically decreased the protein level of ChAT, but not AChE or α7-nAChR. Donepezil prevented isoflurane-induced spatial memory impairments and increased ChAT levels, which were downregulated by isoflurane. In conclusions, pretreatment with the AChE inhibitor donepezil prevented isoflurane-induced spatial memory impairment in aged mice. The mechanism was associated with the upregulation of ChAT, which was decreased by isoflurane.

## Introduction

Nearly 10–20% of elderly patients suffer from postoperative cognitive dysfunction (POCD) within 3–6 months after surgery [1], [2]. Although many authors [3], [4], [5] believe that POCD is self-limited, POCD is still a major clinical issue. Monk [6] demonstrated that patients experiencing POCD are at an increased risk of death in the first year after surgery. Steinmetz [7] showed that POCD after noncardiac surgery was associated not only with increased mortality but also with the risk of leaving the labor market prematurely and dependency on social transfer payments. However, there is currently no clinically available treatment to prevent POCD. Although some studies have not found any differences in the rates of POCD when comparing regional and general anesthesia [4], [5], exposure to general anesthesia is still considered a possible cause of POCD in the elderly [8], [9]. Many studies have shown that volatile anesthetics impair the spatial memory of aged rats [10], [11]; however, the mechanism is not well understood. Although volatile anesthetics have been shown to induce apoptosis in cultured cells [12], [13], there is no evidence to prove the relationship between neural apoptosis and cognitive dysfunction induced by volatile anesthetic exposure in older animals. Theoretically, the anticholinergic properties of drugs are a biologically plausible cause of cognitive dysfunction in elderly subjects. Notably, the anticholinergic drug scopolamine has been shown to induce or exacerbate preexisting cognitive impairment [14]. Moreover, three cross-sectional population-based studies have demonstrated that anticholinergic drug intake is an important factor in determining the severity of cognitive impairment [15], [16], [17]. Because previous studies have shown that volatile anesthetics have anticholinergic properties [18], we hypothesized that pretreatment with the acetylcholinesterase inhibitor donepezil, which has been approved by the Food and Drug Administration (FDA) for the treatment of Alzheimer's disease, may prevent isoflurane-induced spatial memory impairment in aged mice.

## Results

### Donepezil pretreatment prevented spatial memory impairment from isoflurane exposure

**Figure 1:**
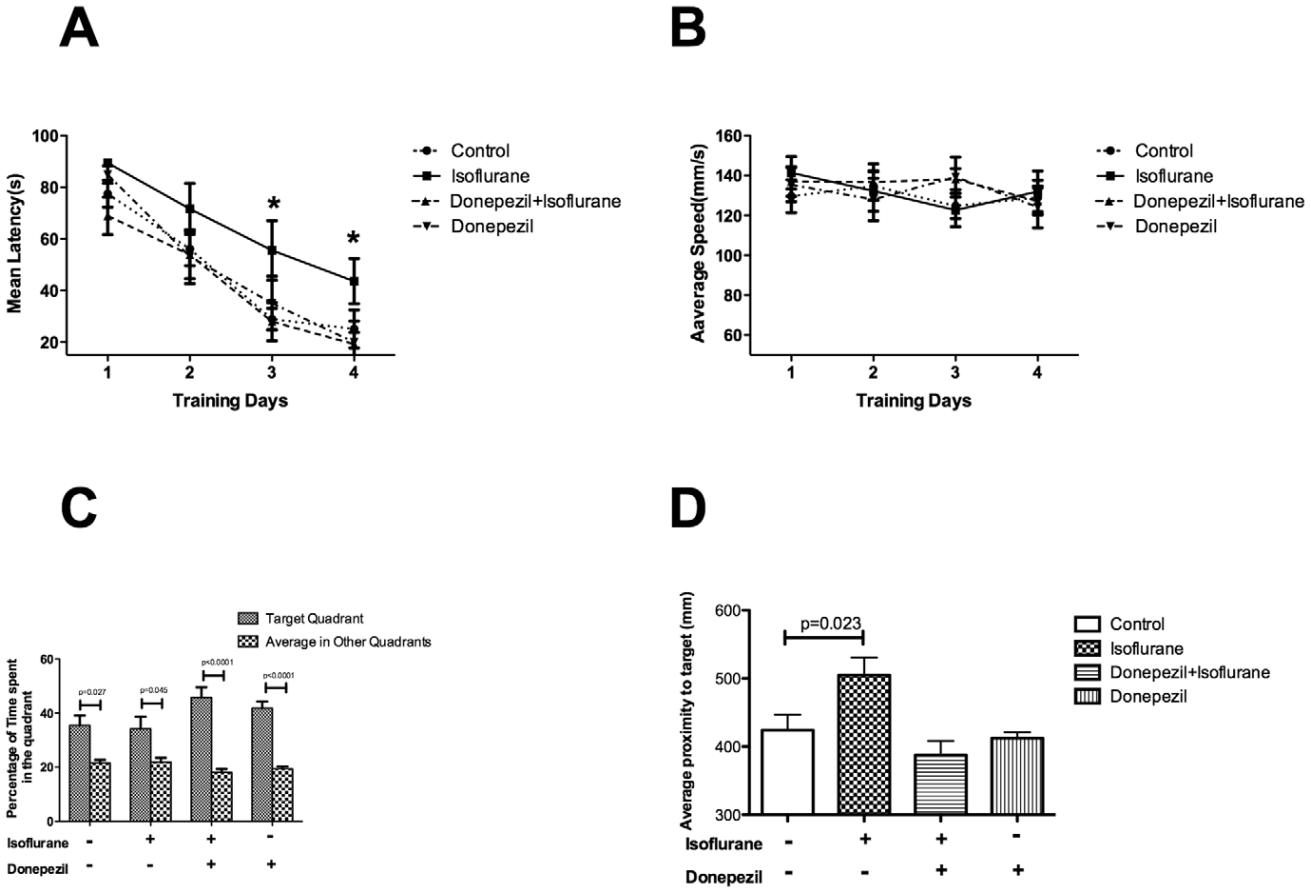
Donepezil pretreatment prevented spatial memory impairment induced by isoflurane exposure. Following pretreatment with donepezil or saline, spatial memory was tested with the Morris water maze two weeks after exposure to isoflurane or the carrier gas. A: In spatial acquisition trials, the mean latency to reach the platform was longer in the isoflurane group compared with the control group (p = 0.04) and the isoflurane+donepezil group (p = 0.008). B: Swimming speed was similar among the four groups. C: In the probe trial, the percentage of time spent in the target quadrant was longer than the average percentage spent in other quadrants (p<0.05) for all four groups, but there were no differences between the groups for the percentage of time spent in the target quadrant. D: The average proximity to the target in the probe test of the isoflurane group was further away than the average proximity in the control group (p = 0.023). N = 12 for each group.

As showed in Figure 1A, the statistical analyses indicate that the repeat factor (days) had significant effects on the latency (p<0.001); the overall group factor also had a significant effect on latency (p<0.05). Post hoc Bonferroni analysis showed that animals in the isoflurane exposure group took longer to find the platform than mice in the isoflurane+donepezil group (p<0.005) and the control group (p<0.05). However, no interactive effect between group and days was found (p>0.05).

Statistical analyses showed that on day three and day four, animals in the isoflurane exposure group took longer to find the platform (day 3, isoflurane exposure vs. control: p<0.05; isoflurane exposure vs. isoflurane+donepezil: p<0.05; day 4, isoflurane exposure vs. control: p<0.05; isoflurane exposure vs. isoflurane+donepezil: p<0.05). All mice appeared to swim normally with similar swimming speed, and none of the mice demonstrated any difficulty in mounting the hidden platform (Fig. 1B).

Although the percentage time spent in the target quadrant was significantly longer than the average percentage time spent in other quadrants for all mice in the probe trial, the time percentage spent in the target quadrant was similar among the four groups (Fig. 1C). However, the average proximity to the target for the mice in the isoflurane group was further away than the average proximity of the mice in the control group (p<0.05); there was no difference between the isoflurane+donepezil group and the control group (Fig. 1D).

### Isoflurane exposure decreased choline acetylase protein levels

**Figure 2:**
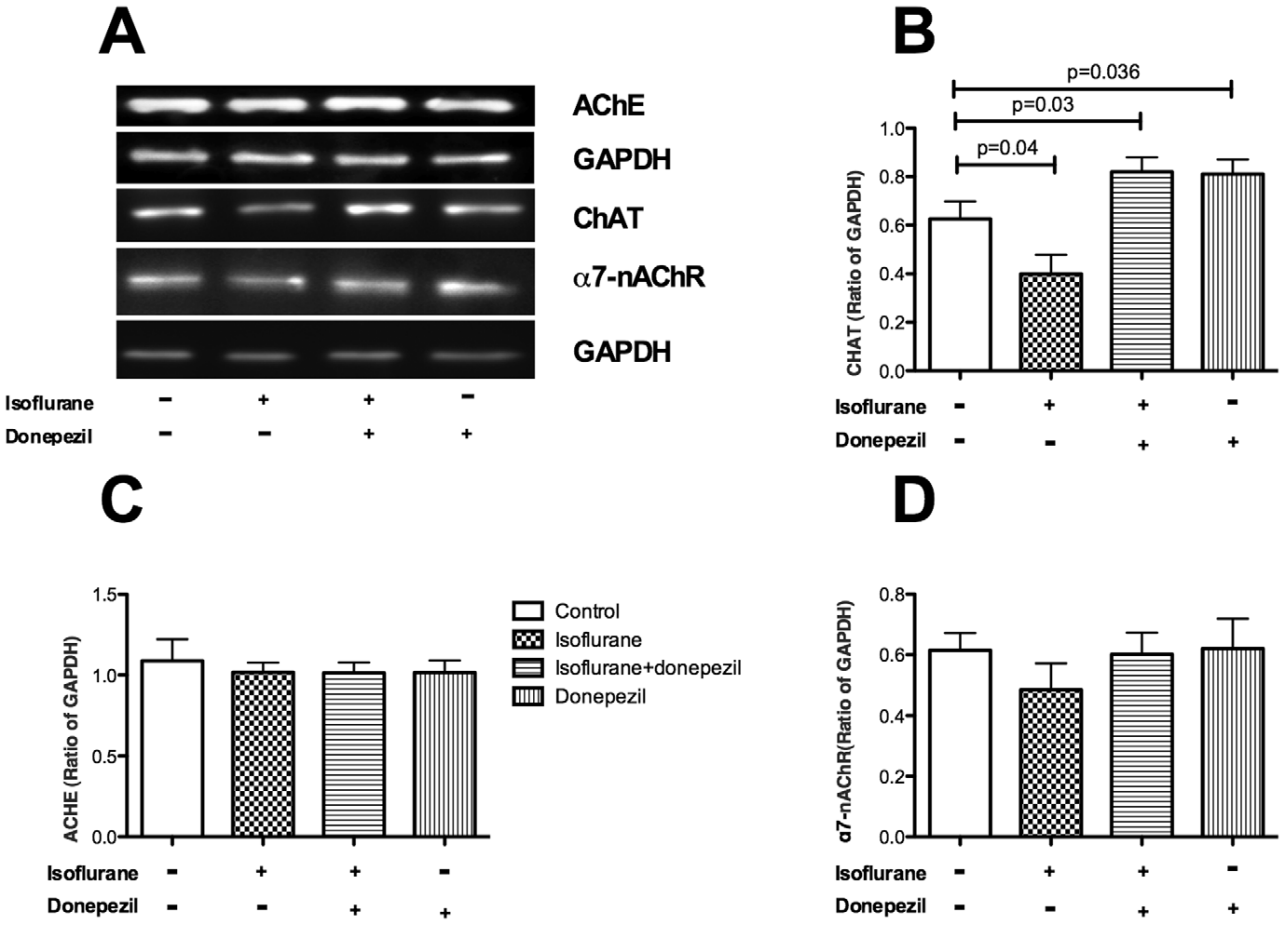
Donepezil pretreatment increased the expression of ChAT which was decreased by isoflurane exposure. A: Isoflurane exposure decreased ChAT levels, and donepezil increased the expression of ChAT, as detected using Western blotting. B: Statistical analyses demonstrated significant differences in ChAT expression among the four groups. C: We did not observe any differences in AChE levels among the four groups. D: In addition, we did not observe any differences in α7-nAChR levels among the four groups. ChAT: choline acetyltransferase; AChE: acetylcholinesterase; α7-nAChR: α7-nicotinic acetylcholine receptor. N = 10 in each group.

Western blotting results showed that isoflurane exposure dramatically decreased ChAT protein levels (p = 0.04). ChAT protein levels were significantly increased in the donepezil and donepezil+isoflurane groups compared to the controls (p<0.05 vs. p<0.05) (Fig. 2A, B). However, there were no differences among the four groups with regard to AChE or α7-nAChR protein levels (Fig. 2A, C, D).

### Hemodynamics and blood gas analysis

**Figure 3:**
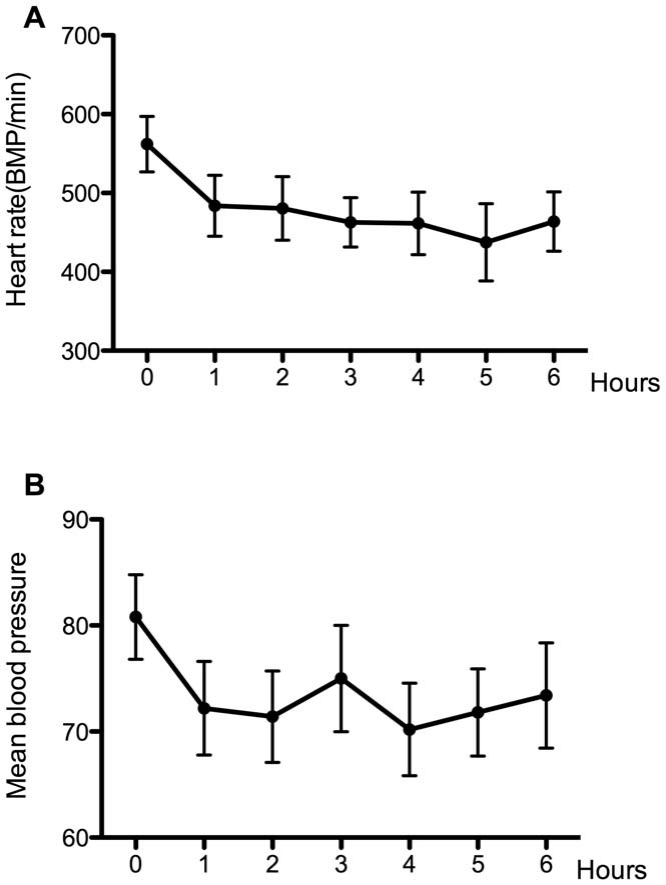
Six-hour isoflurane anesthesia had no significant effects on blood pressure and heart rate. Separate animals were used to monitor blood pressure and heart rate. During the six-hour isoflurane exposure, animals maintained stable heart rates (A) and stable blood pressure (B).

Isoflurane anesthesia may decrease blood pressure and depress respiration; therefore, we monitored blood pressure and heart rate during anesthesia. As shown in Figure 3, blood pressure and heart rate decreased after isoflurane anesthesia, but they remained stable over the course of the anesthesia. To prevent hypoxia during isoflurane anesthesia, we analyzed blood gas at the end of the isoflurane exposure, which revealed normal oxygenation (129.6±1.86 mmHg), adequate PCO_2_ values (40.4±1.53 mmHg) and moderate acidosis (pH: 7.30±0.05).

## Discussion

The major finding of the present study was that pretreatment with the acetylcholinesterase inhibitor donepezil prevented the spatial memory impairment induced by six hours of isoflurane (1.2%) exposure. The mechanism of these protective effects may relate to elevated ChAT levels in the brain. To our knowledge, the present study is the first demonstration that pretreatment with donepezil, which has been approved by the FDA for the treatment of Alzheimer's disease, prevents isoflurane-induced spatial memory impairment in aged mice.

Similar to our study, many other reports have shown spatial memory impairment after isoflurane exposure [11], [19], [20]. Other studies have also demonstrated the anticholinergic effects of isoflurane. Indeed, Grasshoff [21] found that acetylcholine significantly reduced both the potency and efficacy of isoflurane on the potential activity of cortical slices from rats. Furthermore, using cerebral microdialysis, Whittington [22] found that rat hippocampal acetylcholine (ACh) levels decreased to 36.3±13.9% of baseline levels after an 80-minutes exposure to 1 minimum alveolar concentration (MAC) of isoflurane. We demonstrated that levels of ChAT protein, which is the rate-limiting enzyme for the synthesis of acetylcholine, decreased dramatically two weeks after isoflurane exposure.

Donepezil, which is an antagonist of AChE, is a clinically approved medication used to treat Alzheimer's disease patients [23]. In the present study, we show that donepezil can prevent isoflurane-induced spatial memory impairment. This finding suggests new possibilities for its clinical application to treat postoperative cognitive dysfunction.

Lee [24] found that animals that received donepezil (0.3 mg/kg/day, intraperitoneal) had increased ChAT immunoreactivity in the cerebral cortex, which is similar to the present results. We demonstrated that intragastric donepezil (5 mg/kg/day) administration for four weeks increased ChAT levels in the donepezil group and the donepezil+isoflurane group. However, the MWM data indicate that donepezil does not make animals in the isoflurane+donepezil and donepezil groups more clever than the control mice. The mechanism responsible for these effects is unknown. Kakinuma [25] found that ChAT levels in the ventricular myocardium increased after donepezil treatment, which was accompanied by an increase in ChAT promoter activity. More studies should be performed to determine the detailed mechanism for these effects. Although Zivin [26] found that AChE mRNA in the brain increased significantly after 28 days of donepezil treatment (2 mg/kg, subcutaneous), we did not find any changes in AChE protein levels among the four groups.

Alpha7-nAChRs are one of the major functional nAChR subtypes in the brain [27], and these receptors play an important role in learning and memory [28]. Although Takadatakatori [29] showed the upregulation of α7-nAChRs in primary culture rat cortical neurons after chronic donepezil treatment (10 µM, four days), we did not observe any significant changes in α7-nAChRs levels between groups. With the two-electrode voltage-clamp technique, Jackson [22] demonstrated that isoflurane and halothane inhibited acetylcholine-evoked currents (100 µM) of α7-nicotinic acetylcholine receptors in *Xenopus* oocytes in a reversible and concentration-dependent manner. Studies are needed to examine α7-nAChRs after donepezil and volatile anesthetic exposure *in vivo*.

Six-hour isoflurane exposure in 30% oxygen resulted in stable blood pressure and heart rate, normal oxygenation, adequate PCO_2_ and moderate acidosis. Similarly, Szczesny [30] demonstrated that 0.8–1.3% isoflurane exposure for 6.5 hours resulted in a stable mean blood pressure and heart rate in mice. With an oximeter probe, Ewald [31] found that oxygen saturation remained at ∼97% at anesthesia levels of 0.9%–1.25% isoflurane. Because it is quite difficult to acquire a sufficient volume of arterial blood from mice without anesthesia for analysis, there is currently no record of normal blood gas values for mice. For this reason, many studies do not assess blood gases [11], [19], [32], [33]. Therefore, we do not know how to define and evaluate the effects of acidosis in mice. However, in the present study, no animals died after six hours of isoflurane exposure, which indicated that even if some minimum physiological changes occurred, they were of little clinical relevance.

While this study has provided some interesting data, it also has limitations. To observe the long-term effects of isoflurane exposure, we only performed the behavioral and biochemical tests two weeks after isoflurane exposure, as did other studies [11], [34], thus we do not know the acute effects of isoflurane exposure in aged mice. Saab [35] found that in adult mice exposed to 1.3% isoflurane for 1 h, contextual fear memory persisted for 24 hours after isoflurane exposure. Our previous study showed that repeated isoflurane exposure improved spatial memory [36]. Additional studies are needed to demonstrate the acute effects of isoflurane on the aged mice. In the present study, we only performed our behavioral and biochemical tests after donepezil pretreatment and isoflurane exposure; therefore, more studies should be performed to assess the causal relationship between the behavior and alterations in ChAT levels.

The present study used aged mice, but caution should be paid to transferring the preventative effects of donepezil to other subjects. Although we demonstrated that donepezil prevented the isoflurane-mediated decrease in ChAT levels, more studies should be performed before donepezil can be clinically used to treat POCD.

In conclusion, isoflurane (1.2%) exposure for six hours impaired the spatial memory of aged mice. Donepezil prevented the isoflurane-induced impairment, which was associated with increasing ChAT levels that were reduced by isoflurane.

## Materials and Methods

This study was approved by the Shanghai Jiaotong University School of Medicine Animal Care and Use Committee(Permit Number: Renji-09-1010). All procedures were performed in accordance with the guidelines of the National Institutes of Health (NIH) for animal care (Guide for the Care and Use of Laboratory Animals, Department of Health and Human Services, NIH Publication No. 86-23, revised 1985). Every effort was made to minimize suffering and the number of animal used.

### Animals and anesthetic exposure

The present study used 18-month-old mice (C57BL/6J), which were raised in the Animal Research Center of Shanghai Jiaotong University, School of Medicine. All animals were housed in their home cages, and a standard rodent diet and water were available ad libitum. Room temperature was maintained at 22±1°C, and relative humidity was set at 50±10%. Daily body weights were recorded for each animal during the experiment, and no changes were detected throughout the study.

Anesthetic exposure was performed in a chamber that was partially submerged in a circulating 37°C water bath. A total of 30% oxygen and 70% nitrogen flowed (2 L/min) through a calibrated isoflurane vaporizer into the chamber. Oxygen, carbon dioxide and isoflurane concentrations were continually monitored in the effluent gas chamber using infrared absorbance (Ohmeda 5330, Detex-Ohmeda, Louisville, CO). Rectal temperature was measured and maintained at a physiological temperature of 37.0±0.2°C in all groups.

### Experimental protocols

Eighteen-month-old mice were randomly divided into four groups: control, isoflurane, donepezil+isoflurane and donepezil (n = 12 in each group). Mice in the donepezil and donepezil+isoflurane groups received donepezil (5 mg/kg, Pfizer, NY) by oral gavage with a feeding needle in 0.5 ml saline every day for four weeks. Mice in the other two groups received 0.5 ml saline by oral gavage with a feeding needle. Four weeks later, mice in the donepezil+isoflurane and isoflurane groups were exposed to 1.2% isoflurane for six hours. Mice in the donepezil and control groups were exposed to vehicle gas (30% O_2_+ 70% N_2_) for six hours. Two weeks after anesthesia exposure, mice were subjected to the Morris water maze (MWM) to evaluate spatial memory. Following the MWM test, the mice were sacrificed, and brains were removed for Western blot quantitation of acetylcholinesterase (AChE), choline acetylase (ChAT) and α7 nicotinic receptor (α7-nAChR) protein expression.

### Hemodynamic monitor and blood gas analysis

Isoflurane (1.2%) anesthesia was administered for six hours by a mask that had been specifically designed for use with mice (n = 5). Blood pressure and heart rate were measured before and during anesthesia (every 30 minutes) with a noninvasive blood pressure meter (Softron, Beijing, China). At the end of anesthesia, the each animal's abdomen was quickly opened, and the abdominal aorta was exposed. With a 24G venal catheter, 0.5–1 ml blood was drawn for blood gas analysis.

### Morris water maze

The Morris water maze trials were performed by a person blinded to the group conditions, as previously described [37], [38]. The water maze consisted of a painted circular pool (110 cm diameter and 30 cm high) in which mice were trained to escape from the water by swimming to a hidden platform (1.5 cm beneath the water's surface), the location of which could only be identified using distal extra-maze cues attached to the room walls. Water was kept at 20°C and opacified with titanium dioxide for all training and testing. The pool was divided into four quadrants: north (Target), south (Opposite), east (Adjacent 1) and west (Adjacent 2). The experiments were recorded using a camera connected to a video recorder and a computerized tracking system.

The MWM testing began on the 15^th^ day after isoflurane exposure and continued for five days. The first four days were used for the spatial acquisition phase, which consisted of a total of 16 training trials (four training trials per day and four training days with an inter-trial interval of 20 minutes). At the beginning of each trial, the mouse was placed into the water facing the wall of the pool at one of the four quadrants. Although the starting point was randomly selected, the protocol was fixed at the beginning of each trial and was maintained throughout all four acquisition trials. Each mouse was allowed 90 s to find and mount the platform. Thirty seconds after the mouse had mounted the platform; the mouse was removed, placed in a holding cage and warmed with a heating lamp. Mice that failed to locate and mount the platform within 90 s were placed on the platform for 30 s before they were transferred to the holding cage. A video camera mounted above the pool was used to track the mice. The amount of time spent finding and mounting the platform (escape latency) and swimming speed were calculated from the recorded videos using MWM software (Shanghai Jiliang Software Technology Co. Ltd., China).

On the fifth day (the final day), the mice participated in a probe trial in which the platform was removed. Mice were allowed to swim freely for 60 s, and the percentage of time spent in the target quadrant and the average percentage spent in other quadrants were recorded. Each individual animal may use a different strategy for looking for the platform during the probe trial; therefore, we calculated the average proximity to the former location of the platform from the recording. Gallagher [39] showed that average proximity to the target was a more sensitive measure than the time spent in the target quadrant for Long–Evans rats. Magnusson [40], [41] used the average proximity to measure the spatial memory of aged mice. Sprague [42] used the proximity score calculated from proximity to evaluate the memory of aged rats. Their results demonstrated that proximity was suitable to test the spatial memory of aged rodents.

### Western blot analyses

After mice were euthanized using carbon dioxide, their brains were quickly removed. Each brain was homogenized in RIPA lysis and extraction buffer (25 mM Tris-HCl, pH 7.6, 150 mM NaCl, 1% NP-40, 1% sodium deoxycholate, 0.1% SDS), which contained a mixture of phosphatase inhibitors and protease inhibitors. Protein concentrations were determined using a protein assay kit (Bio-Rad, Hercules, CA). Protein samples were dissolved in 2× sample loading buffer, denatured at 95°C for 5 minutes and separated by sodium dodecyl sulfate-polyacrylamide gel electrophoresis (SDS-PAGE) on a 10% gel. An equal amount of protein from each sample was transferred electrophoretically to nitrocellulose membranes (Amersham Biosciences, Piscataway, NJ). The membranes were blocked with 5% milk in Tris-buffered saline and Tween 20 (TBST) for 1 h at room temperature and incubated overnight at 4°C with rabbit anti-acetylcholinesterase (AChE) antibody (1∶500, Santa Cruz Biotechnology, Inc., Santa Cruz, CA), goat anti-choline acetyltransferase antibody (1∶800, Abcam, Cambridge, MA), rabbit anti-nicotinic acetylcholine receptor α-7 antibody (1∶800, Abcam, Cambridge, MA) or rabbit anti-actin antibody (1∶5,000, Cell Signaling, Danvers, MA) in TBST plus 1% bovine serum albumin (BSA). After incubation in primary antibody, the blots were incubated for 1 h at room temperature with the appropriate horseradish peroxidase (HRP)-conjugated secondary antibody (anti-rabbit or anti-goat; 1∶5000, Santa Cruz Biotechnology, Inc.) prior to detection with an enhanced chemiluminescence (ECL) system (Pierce Biotechnology, Rockford, IL). The membranes were then exposed to X-ray film. Four samples from each group were examined (n = 4). ImageJ (NIH free software) was used to compare the band densities.

### Statistics

All data are presented as the mean ± SEM. Statistical Package for the Social Sciences (SPSS) v.10.0 software was used for statistical analyses. Data obtained using Western blotting were analyzed with one-way analysis of variance (ANOVA) followed by a least square difference (LSD) multiple comparison test. Behavioral studies were analyzed using two-way ANOVA with repeated measures (anesthesia treatment as the between groups factor and time as the repeated measures factor) followed by Bonferroni multiple comparison test to compare the four groups. Differences were considered statistically significant at p<0.05.
